# Functions of Fasciculation and Elongation Protein Zeta-1 (FEZ1) in the Brain

**DOI:** 10.1100/tsw.2010.151

**Published:** 2010-08-17

**Authors:** Andrés D. Maturana, Toshitsugu Fujita, Shun'ichi Kuroda

**Affiliations:** ^1^ Department of Bioengineering, Nagaoka University of Technology, Niigata, Japan; ^2^ Research Institute for Microbial Diseases, Osaka University, Osaka, Japan; ^3^ Graduate School of Bioagricultural Sciences, Nagoya University, Chikusa, Nagoya, Japan

**Keywords:** FEZ1, neuronal differentiation, neuronal disorders, virus infection, organelle transport

## Abstract

Fasciculation and elongation protein zeta-1 (FEZ1) is a mammalian ortholog of the *Caenorhabditis elegans* UNC-76 protein that possesses four coiled-coil domains and a nuclear localization signal. It is mainly expressed in the brain. Suppression of FEZ1 expression in cultured embryonic neurons causes deficiency of neuronal differentiation. Recently, proteomic techniques revealed that FEZ1 interacts with various intracellular partners, such as signaling, motor, and structural proteins. FEZ1 was shown to act as an antiviral factor. The findings reported so far indicate that FEZ1 is associated with neuronal development, neuropathologies, and viral infection. Based on these accumulating evidences, we herein review the biological functions of FEZ1.

## DISCOVERY OF FASCICULATION AND ELONGATION PROTEIN ZETA-1 (FEZ1)

FEZ1 is a mammalian ortholog of UNC-76, a protein found in the nematode *Caenorhabditis elegans.* UNC-76 has been used in an attempt to elucidate the mechanisms of locomotory defects. Genetic screenings of *C. elegans* mutants showing locomotory defects (uncoordinated or *unc* mutants) allowed the identification of various genes related to deficiencies in axonal guidance. Based on such screening, at least two groups of genes were found to be necessary for axonal elongation in fascicles[1,2,3]. The first group (*unc-14, unc-33, unc-44, unc-51*, and *unc-73*) is required for axonal elongation along non-neuronal cell surfaces, as well as neuronal cell surfaces. The second group (*unc-34, unc-71*, and *unc-76*) is essential for the elongation of axons in fascicles, but not along non-neuronal cell surfaces. This suggests that these genes encode proteins essential for the interaction of axons with neuronal surfaces.

The *C. elegans unc-76* mutant exhibits two types of axonal defects in fascicles[3,4]. First, the extension of axons from the hermaphrodite-specific neuron (HSN) does not reach the length of wild-type worms. Second, the axons of the dorsal and ventral D-neurons (γ-aminobutyric acid [GABA]–containing neurons) fail to bundle tightly together. Therefore, UNC-76 is considered to be essential for the elongation of axons and the formation of nerve fiber bundles during neuronal development. Around the same time, FEZ1 was cloned as a mammalian ortholog of UNC-76. Transformation of *C. elegans unc-76*

mutant germlines with the human *FEZ1* cDNA, placed under the control of the 1-kb fragment of *C. elegans unc-76* promoter, partially restores the normal locomotion and fasciculation of the GABA-containing neurons of the ventral nerve cord[4]. However, FEZ1 expression in humans does not rescue the outgrowth of HSN axons. These observations indicate that nematode UNC-76 and human FEZ1 share functional similarity, but they possess distinct structural and functional properties.

## MOLECULAR ORGANIZATION AND EXPRESSION PROFILE OF FEZ1

In the human genome, the *FEZ1* gene is located on chromosome 11. The human *FEZ1* gene encodes a protein of 392 amino acids with a molecular mass of 45 kDa, showing ~35% identity with UNC-76. Fig. 1 represents a schematic structure of the human FEZ1 protein. The protein harbors four regions of amphipathic helices constituting coiled-coil structures that are utilized for intra- and intermolecular interactions[5]. Indeed, under the *in vitro* condition, FEZ1 forms a homodimer or a heterodimer with FEZ2, a homolog of FEZ1 (see below). The dimerization is mediated by a coiled-coil region in the C-terminal half-domain[6,7]. However, the *in vivo* significances of the dimerization have yet to be elucidated. As described below, FEZ1 interacts with various signaling, structural, and functional molecules (Fig. 2). The identified interactions with various proteins are mediated by a region encompassing one or more of the coiled-coil domains (Fig. 1). Therefore, multiple coiled-coil domains in FEZ1 act as motifs for dimerization and association with other proteins. FEZ1 may then serve as an adaptor or scaffold protein, allowing the assembly of other proteins into a signaling or functional complex.

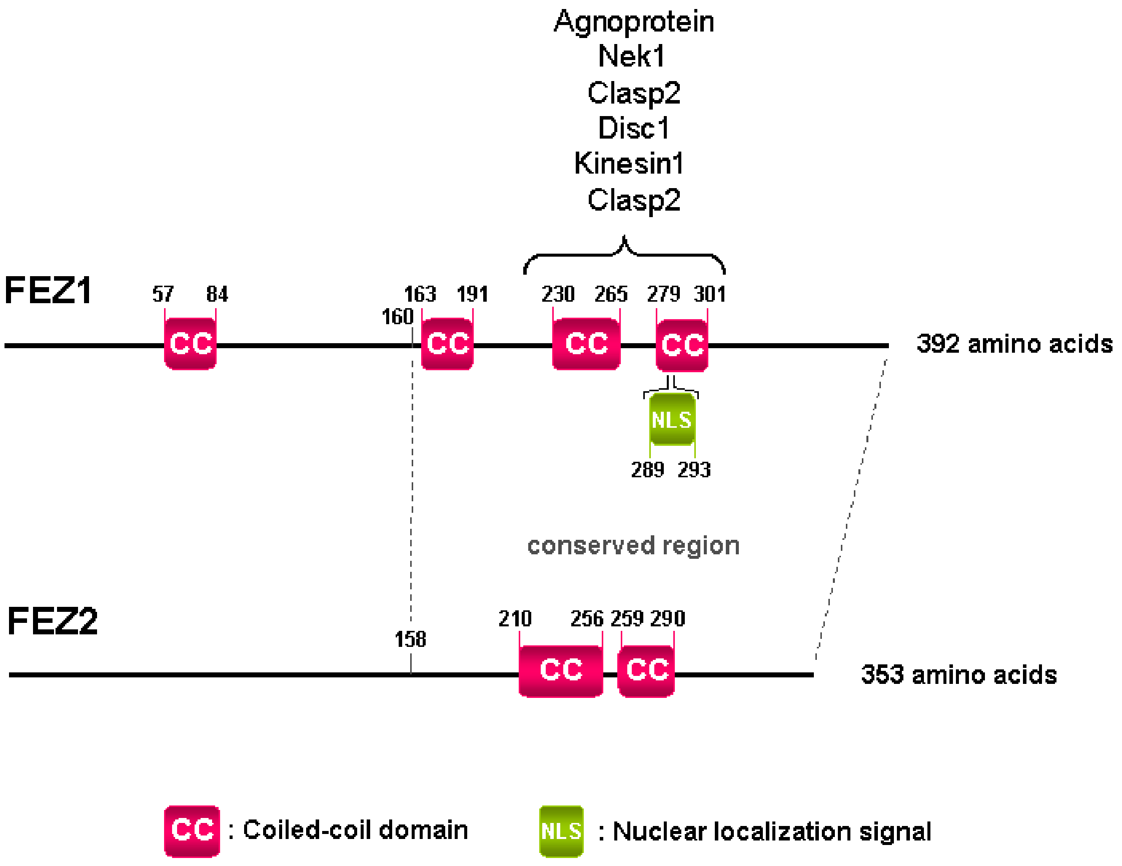

**FIGURE 1.** Schematic structures of human FEZ1 (GenBank accession number: AAC51282) and FEZ2 (GenBank accession number: BAD06207). Curly bracket indicates the region of FEZ1 involved in interaction with several proteins.

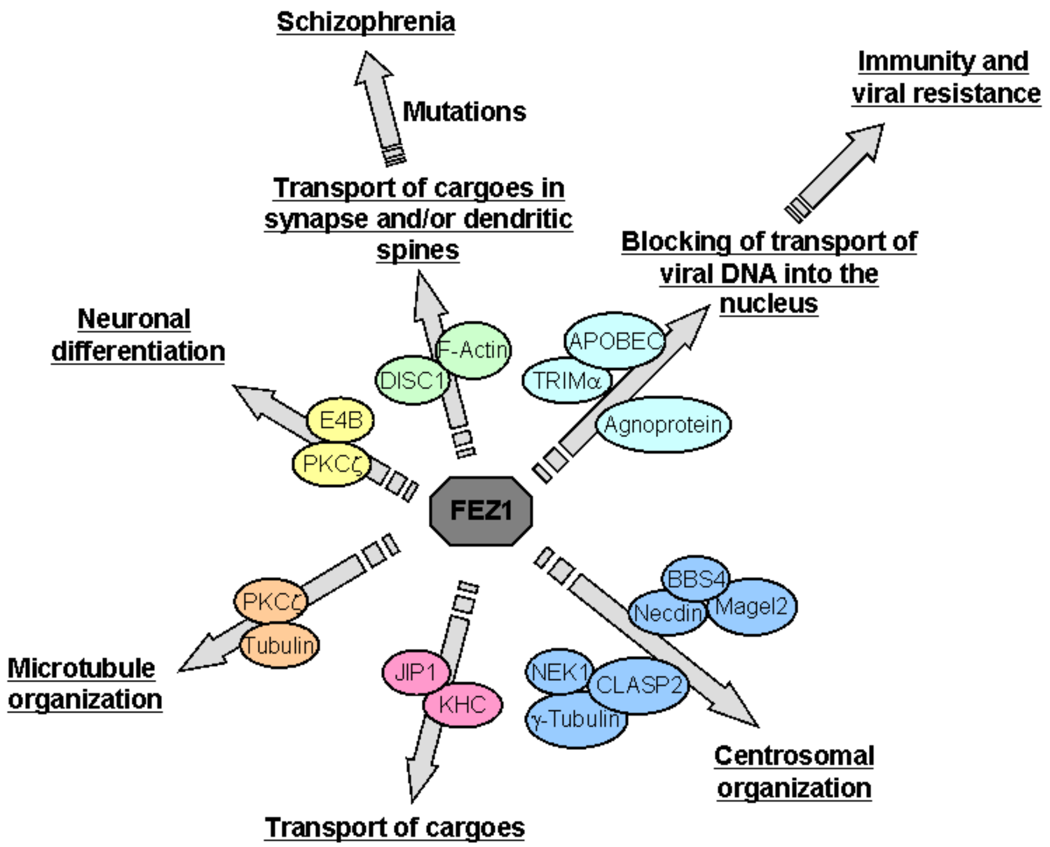

**FIGURE 2.** Functions of FEZ1 based on its binding partners. Details are described in the text.

In addition to multiple coiled-coil domains, the carboxyl (C)-terminal half-region of FEZ1 contains a nuclear localization signal (NLS)[8]. FEZ1 undergoes post-translational modifications that may regulate its unidentified molecular functions: phosphorylation by protein kinase C ζ (PKCζ)[5,7], ubiquitination by E4B enzyme[9], and *N*-glycosylation at four potential N-glycosylation sites[5].

In adult rats, *FEZ1* mRNA is exclusively expressed in the brain. In particular, abundant expression is seen in the mitral and granular cells of the olfactory bulb, the granule cells of the dentate gyrus region CA1-3 in the hippocampus (highest expression)[10], and in the Purkinje and granular cells of the cerebellum[6,11]. An immunofluorescence analysis showed that FEZ1 protein is mainly expressed in GABAergic inhibitory neurons, the axon bundles of the granule cells in the dentate gyrus[10], and astrocytes[12]. In adult mouse brains, *FEZ1* mRNA and FEZ1 protein were also detected in similar regions[10]. During the embryonic stage of the mouse, the expression level of *FEZ1* mRNA gradually increases during brain development, with a maximum level 11 days *post coitum*[6,11]. Thus, the expression of *FEZ1* mRNA increases during the development of the nervous system of the mouse. The peak of FEZ1 expression at day 10 corresponds to the period of axonal outgrowth: day 9.5 to day 12.5[13]. At the subcellular level, the FEZ1 protein is localized in the cell body and neurites of rat pheochromocytoma PC12 cells[14], and in the axons and dendrites of hippocampal neurons[15].

When expressed in human embryonic kidney HEK293 cells, FEZ1 is localized not only in the cytoplasm, but also in the nucleus[8]. This result can be attributed to the presence of NLS in the C-terminal half-region (see above). Moreover, Lanza et al.[8] reported that FEZ1 localizes at the centrosome through the interaction with γ-tubulin. The localization of FEZ1 in the nucleus suggests a role in transcriptional regulation or chromatin remodeling. Indeed, yeast two-hybrid screening has shown that FEZ1 interacts with various transcriptional regulating proteins and chromatin remodeling proteins (e.g.,

BAF60a, Zinc finger protein 253, DRAP1, Tlk2)[7]. Moreover, the NLS is located within the C-terminal coiled-coil domain. The binding of a protein in this region may therefore mask the signal for translocation to the nucleus. The functions of FEZ1 in the nucleus have yet to be investigated.

## BINDING PARTNERS AND BIOLOGICAL FUNCTIONS OF FEZ1

The biological functions of FEZ1 would be involved in the development and function of the central nervous system because of its brain-specific expression. Using proteomic approaches (particularly yeast two-hybrid screening assays), various functional proteins have been identified to interact with FEZ1, including factors that have roles in the cytoskeleton network, transport of cargoes, regulation of the cell cycle, and retrovirus propagation. Thus, the diversity of these binding partners implies that FEZ1 is a multirole molecule in neuronal development, neuropathologies, and neuronal resistance to viral infection (Fig. 2).

### Neuronal Differentiation through Phosphorylation by PKCζ

FEZ1 was identified as a new PKCζ-interacting protein[5] by yeast two-hybrid screening of a rat brain–derived cDNA library using the regulatory domain of PKCζ as bait. Under the *in vitro* condition, PKCζ efficiently phosphorylates FEZ1. Coexpression of FEZ1 with a constitutively active mutant of PKCζ in PC12 cells stimulates its neuronal differentiation[5]. Knockdown of FEZ1 expression with *FEZ1* small interfering RNA (siRNA) prevents the neuronal differentiation of nerve growth factor (NGF)–treated PC12 cells[14] and the growth of axons (but not dendrites) in cultured hippocampal neurons[15]. Thus, FEZ1 phosphorylated by PKCζ would give neuronal cells the “cue” for their differentiation. By another yeast two-hybrid screening, FEZ1 was identified as an interacting protein of the U-box-type ubiquitin protein isopeptide ligase E4B[9]. E4B mediates the polyubiquitination of FEZ1, which is not a signal of degradation, but a functional post-translational modification for FEZ1. Moreover, a dominant-negative mutant of E4B inhibits the neuronal differentiation induced by the coexpression of FEZ1 and PKCζ[9]. However, how the phosphorylated FEZ1/E4B complex is involved in neuronal differentiation at the molecular level is incompletely understood.

### Regulation of Anterograde Axonal Transport by the Interaction with the Kinesin Superfamily

In an attempt to discover new interacting proteins for *Drosophila* motor protein kinesin-1, the C-terminus of kinesin heavy chain (KHC) was identified to interact with *Drosophila* UNC-76 by genetic analysis[16]. The loss of function of *Drosophila* UNC-76 results in a defect of axonal transport, leading to lethality at the transition between the second and third instar at 5 days of embryonic development. Similar phenotypes were also observed in *kinesin* mutants, suggesting a crucial role of UNC-76 in kinesin-dependent axonal transport. Consistently, FEZ1 was found to bind to KIF5 (a mammalian counterpart of kinesin-1) in rat hippocampal neurons[14]. Additionally, Blasius et al.[17] reported that a kinesin-1/FEZ1 complex includes JNK-interacting protein 1 (JIP1) for appropriate transport of cargoes along microtubules. In this machinery, FEZ1 would cooperate with JIP1 to activate the motility of kinesin-1 as well as its binding activity to microtubules[17]. Consistent with the anterograde transport mediated by kinesin-1, *FEZ1* silencing using siRNA strongly reduced the anterograde (but not retrograde) movement of mitochondria in the neurites of NGF-treated PC12 cells, as well as in the axons of rat hippocampal neurons[14,15]. In fact, FEZ1 could not interact with dynein, a retrograde motor protein[14]. Taken together, FEZ1 would appear to be indispensable for anterograde transport in neurites and axons.

## Stabilizing and Extension of the Microtubule Network

FEZ1 associates with α- and β-tubulin, which comprise the microtubules in extending neuronal axons[18] and extending neurites of NGF-treated PC12 cells[14]. Recently, PKCζ was shown to play an essential part in the remodeling of microtubules during neurite extension[19]. Therefore, FEZ1 may participate in the formation of microtubules in differentiating neurons, presumably through the complex formation of FEZ1, PKCζ, and α-/β-tubulin. The complex may stabilize and extend the microtubule network in parallel with the axonal transport of cargoes.

## Centrosomal Organization by Association with Centrosomal Proteins

Recently, FEZ1 was found to interact with never in mitosis gene A (NIMA)–related kinase 1 (NEK1)[20]. NEK1 is a member of the Ser/Thr-specific kinase family and is involved in cell cycle control at the G2/M checkpoint[21]. Mutations in the *NEK1* gene cause polycystic kidney disease[22]. Coiled-coil regions in the regulatory domain of NEK1 bind to that of FEZ1[23]. Both proteins form a hetero-oligomeric complex colocalizing at the centrosomal region together with γ-tubulin and cytoplasmic linker-associated protein 2 (CLASP2), which is involved in organization of the mitotic spindle and alignment of the kinetochore during cell division[23]. Additionally, FEZ1 was shown to bind to three other proteins (Necdin, Magel2, Bardet-Biedl syndrome 4 [BBS4]) at or near the centrosome[24]. *Necdin* and *Magel2* genes are inactivated in patients with Prader-Willi syndrome, a chromosome-deletion disorder characterized by neonatal hypotonia, developmental delay, hypoventilation, and hypogonadism[25]. The interaction of FEZ1 with Necdin and Magel2 prevents their degradation[24]. Collectively, FEZ1 may participate in the regulation of centrosomal organization by anchoring with various components of the centrosome[23,24].

## FEZ1 in Neuronal Disorders

FEZ1 was found to be an interacting partner of the schizophrenia-related protein disrupted in schizophrenia 1 (DISC1)[26]. The *Disc1* gene is genetically linked to schizophrenia. The first discovery came from a linkage analysis in a Scottish family with a multitude of mental illnesses. Translocation of the *Disc1* gene was found in several members of this family. Several other genetic studies have also shown the link between *Disc1* and schizophrenia[27]. DISC1 is also involved in the development of the cerebral cortex[28]. FEZ1 and DISC1 colocalize in the growth cones of cultured hippocampal neurons, where they interact with F-actin[26]. FEZ1 and DISC1 also cooperate for the neuronal differentiation of PC12 cells[26]. Thus, the molecular interaction of FEZ1 with DISC1 suggests a tight relationship with neuronal pathology. A genetic association between some single nucleotide polymorphisms of *FEZ1* and schizophrenia patients was also reported[29]. However, this association was not confirmed in other case studies[30,31].

More recently, FEZ1-deficient mice were generated[10]. Unexpectedly, the mice did not show any major abnormality in brain development. However, the mice presented abnormal behaviors, including a hyperlocomotion phenotype and enhanced responsiveness to psychostimulants. These abnormal behaviors may have resulted from altered release of dopamine in the mesolimbic pathway of the brain. FEZ1 is expressed in GABAergic neurons[10], so FEZ1 deficiency in these neurons may alter dopaminergic transmission, resulting in abnormal behaviors. In cultured hippocampal neurons, the silencing of FEZ1 by *FEZ1* siRNA inhibits axonal elongation[15]. However, this shortage of axonal length was not observed in FEZ1-deficient mice because an anatomic abnormality in the central nervous system was not observed in the mice[10]. The difference between *in vitro* observations[14,15] and those in FEZ1-null mice could be explained by the presence of FEZ2. Indeed, FEZ2 is also expressed in the brain[6] and could (at least in part) compensate for FEZ1 deficiency. Another possible explanation could be the existence of another yet-unidentified protein compensating for the lack of FEZ1. Also, a technical reason could be that RNAi treatment has a transient effect that can be different from germline manipulation. Further investigation will be necessary in order to understand the role of FEZ1 in the development and diseases of the brain.

## Antiviral Infection Activity

Interestingly, FEZ1 was identified as an interacting partner of agnoprotein, a small protein composed of 71 amino acids and encoded in the JC virus (JCV) genome[18]. JCV is a causative agent for the fatal demyelinating disease-progressive multifocal leukoencephalopathy[32]. Although the functions of agnoprotein are incompletely understood, the small protein has roles in different stages of viral infection[32]. FEZ1 was found to inhibit the propagation of JCV in glial cells, as well as the expression of agnoprotein and capsid VP1 protein in infected cells[18].

Intrinsic immunity (an antiviral system of host cells) is mediated by various intracellular factors that specifically block the infection of eukaryotic retroviruses, such as tripartite motif 5α (TRIM5α) and apolipoprotein B mRNA-editing catalytic polypeptide (APOBEC). Both proteins are responsible for the intrinsic immunity of various cell types (particularly neurons)[33]. TRIM5α recognizes the viral capsid to block viral uncoating and reverse transcription[34]. One family of APOBEC proteins is cytidine deaminase, which induces mutations in the viral genome during reverse transcription. Genetic screening of the mutations that confer resistance to retroviral infection in a fibroblast cell line identified FEZ1 as an antiretroviral infection-resistant factor[35]. The antiviral effect of FEZ1 occurs between the nuclear entry of viral RNA and reverse transcription[36].

The brain is one of the main targets for human immunodeficiency virus type 1 (HIV-1). Most patients infected with HIV-1 are subject to neurological disorders, such as cognitive impairment and HIV-associated dementia[37]. Conversely, neurons are basically resistant to HIV infection because they are deficient in the HIV major receptor CD4 and express intrinsic immunity factors, such as APOBEC3G, a protein suppressing HIV-1 replication[38]. In addition to these proteins, FEZ1 can suppress the infection of retroviruses (particularly HIV-1) in neurons[36], suggesting that FEZ1 takes part in the neuronal resistance to HIV-1 infection.

## Other Functions

Yeast two-hybrid screening of a human fetal brain cDNA library identified 16 FEZ1-interacting proteins[7]. These proteins, containing some of the molecules described above, can be categorized into four groups: (1) six nuclear proteins controlling gene transcription and chromatin organization; (2) five proteins involved in the regulation of neuronal development, microtubule dynamics, and tuning of motor protein activity; (3) two proteins having roles in apoptosis or tumorigenesis; and (4) three proteins whose functions remain unknown. How these FEZ1-originated interactions are involved in the molecular mechanisms of brain functions remains to be elucidated.

## FEZ2

A homolog of FEZ1 (named FEZ2) was first found by Bloom and Horvitz[4]. The human *FEZ2* gene encodes 353 amino acids (Fig. 1)[6]. Human FEZ2 shows 49% identity with human FEZ1 and 34% identity with UNC-76 at the overall amino acid sequence level. The C-terminal halves of FEZs (FEZ1, 169-352; FEZ2 158-353) are highly conserved, although the N-terminal halves have no similarity. Interestingly, *FEZ2* mRNA is ubiquitously expressed and hardly changes during embryonic development[6]. The FEZ2-binding partners reported so far are PKCζ[6] and NEK1[39]. The function of FEZ2 has rarely been investigated, but the coexpression of FEZ2 with a constitutively active mutant of

PKCζ induces the spontaneous neuronal differentiation of PC12 cells[6]. Hence, the biological roles of FEZ2 would be similar to FEZ1. FEZ2 may function for the development of non-neuronal cells by molecular mechanisms similar to those for FEZ1. Additional studies on FEZ2 will be necessary in order to shed light on the relationship with FEZ1.

## CONCLUSIONS AND FUTURE PERSPECTIVES

Despite its restricted expression in the brain, FEZ1 can associate with various proteins at different locations in neurons. The possible functions of FEZ1 are summarized in Fig. 2. The remarkable motifs in FEZ1 are four coiled-coil domains and one NLS. Therefore, using the coiled-coil domains in a spatiotemporal manner, FEZ1 may assemble different molecular complexes by tethering various partners. FEZ1 may act as a scaffolding protein for signaling cascades and the bridging of structural proteins with the transport of cargoes in neurons. Conversely, FEZ1-deficient mice reflect neither the *in vitro* observations nor *unc-76* mutants of *C. elegans*. To understand this discrepancy, characterizing the neurons of FEZ1-deficient mice *in vitro* at the cellular and molecular levels is necessary. The fact that FEZ1 is related to the prevention of viral infection may lead to the development of novel antiviral drugs, although the molecular mechanism remains to be elucidated.

## ACKNOWLEDGMENT

We thank Professor Koichi Takimoto for his critique of the manuscript.
